# Relationship and career challenges faced by people infected with HIV in Malaysia

**DOI:** 10.12688/f1000research.21079.3

**Published:** 2020-10-23

**Authors:** Tuan Norbalkish Tuan Abdullah, Ruhani Mat Min, Mosharaf Hossain, Siti Salina Abdullah

**Affiliations:** 1 Faculty of Business, Economic and Social Development, Universiti Malaysia Terengganu, Kuala Terengganu, Terengganu, 21030, Malaysia

**Keywords:** people affected with HIV, challenges, qualitative research, social constructivist, grounded theory

## Abstract

**Background: **In Malaysia, there are still lack of studies  related to the challenges of people infected with HIV (PIWH). Therefore, this study was conducted to explore the experiences of PIWH and how they cope with HIV.

**Methods:** This qualitative study was based on a social constructivist and grounded theory approach. A total of 12 PIWH were selected by purposive sampling, all of whom participated in semi-structured and audio-recorded interviews, which were supported with non-participant observations and diary entries on three occasions for each participant. The interviews and diaries were transcribed and analysed using the grounded theory approach, which was assisted by utilizing NVIVO-8 to identify the themes related to the experiences of the participants.

**Results: **PIWH experienced challenges related to their career and relationships with family and others. These challenges led to difficulties in gaining employment and career development, as well as feelings of denial, being uncomfortable, rejection, and labelling. They found that their lives were totally and dramatically changed after being tested positive for HIV.

**Conclusions:** Among PIWH, HIV impacted relationships with significant others and career development. The absence of support and acceptance from significant others affected the ability of PIWH to cope with their daily challenges. The results of this study have implications for policymakers in terms of gaining sufficient knowledge and awareness to provide prevention programmes for HIV/AIDS.

## Introduction

The global number of new human immunodeficiency virus (HIV) infections continues to decline. New infections across all ages, as demonstrated by modelled estimates, declined from 3.4 million in 1996 to 1.8 million in 2017; however, progress towards the 2020 milestone of less than 500,000 new infections is far slower ^1^. An estimated 47% of new infections occurred among key populations: men who have sex with men (MSM); people who inject drugs (PWID); people in prisons and other closed settings; sex workers and their clients; transgender people (TG); and the partners of people within these populations. In 2017, globally, there were roughly 21.7 million people living with HIV and receiving antiretroviral therapy (ART) ^2^. In Malaysia, the first reported cases of HIV/AIDS were in 1986, where PWID were largely driving the country’s epidemic. However, the Ministry of Health Malaysia (2018) indicated that the ratio of Malaysian people infected by HIV has declined from 4 in 2000 to 0.2 in 2015 ^3^. The same report also described the populations most affected by the epidemic, with infection rates exceeding 5% among PWID, female sex workers (FSW), TG, and MSM populations. Among the FSW, TG and MSM populations in Malaysia, in 2017, the HIV prevalence was highest (25%) for MSM ^3^.

Although UNAIDS reported that HIV infection has no cure ^4^, PIWH and those at substantial risk can enjoy healthy, long, and productive lives through the use of effective antiretroviral (ARV) drugs to control the virus and prevent transmission ^2^. HIV is also recognized as a highly stigmatized disease ^5^. It challenges an individual physically, socially, and psychologically. Moreover, it can also threaten one’s sense of meaning, purpose, and significance in life ^6^. PIWH may find it difficult to face the reality that they are infected with HIV ^7^, which can contribute to self-denial ^8^ and changes in behaviour ^9^.

HIV/AIDS can affect the physical, social, economic, and psychological condition of the patient. In relation to this, PIWH may encounter numerous problems, such as discrimination, losing social status and role, changes in the patterns of intimate relationships, losing jobs and financial resources, and problems with acquiring the necessary medication ^10^. Many of these problems are common among patients with other chronic diseases, but the stress associated with social and family problems arising from the disease, such as social stigma and exclusion, especially from family and friends, is intensely and uniquely threatening to people with HIV/AIDS ^11^.

The issue of poor relationships can be related to Adler’s concept of social interest as it refers to the awareness of individuals of being part of the human community and the attitude to dealing with the social world. This concept involves the capacity to cooperate and contribute to something bigger than oneself ^12^. The problems faced by PIWH, such as being stigmatized and excluded by others, may contribute to their denial of the infection with HIV, as well as impact their engagement with society ^12^. In relation to this, PIWH may face criticism, stigma, social discrimination, and ruptures in relationships and life projects ^13^. These negative responses can cause social mortality ^14^, as well as have an impact on their physical and mental health ^15^.

A previous study informed that members of both the community and social network may fear being infected with HIV and be frightened of taking care of HIV/AIDS patients ^16^. The stigma or negative views from society can lead to the isolation and low self-esteem among PIWH until they feel depressed, which can contribute to feelings of inferiority ^17^. In contrast, support and positive relationships with others can be sources of motivation to strive for skills, success, and the completion of their goals, which, ultimately, might convert the feeling of inferiority to that of feeling positive ^12^. Failure to notice and concentrate on patients’ problems may lead to lower levels of accountability and an increase in the pessimism of the infected persons towards society, and thus, lead to the further spread of the virus ^10^.

However, as there has been a lack of research concerning the experience of challenges faced by PIWH based on the mode of HIV transmission, this study examines different experiences and challenges among PIWH participants. The main purpose of this study is to understand the experiences of PIWH living with this disease from their own perspectives. This research will explore the experiences of PIWH, with a focus on their lives after being infected with HIV. Qualitative inquiry will be used to capture the challenges and experiences, as well as to allow for any new themes or ideas to emerge. To the researcher’s knowledge, this is the first study to explore these experiences among PIWH based on the mode of HIV transmission.

## Methods

This qualitative study using a social constructivist approach was conducted between March 2017 and February 2018 at two selected public hospitals in Malaysia (Hospital Sultanah Nur Zahirah and Hospital Sungai Buloh). Ethical approval to conduct the study was obtained from the Medical Research and Ethics Committee of the Ministry of Health Malaysia KKM/NIH/P17-93, dated 28 ^th^ February 2017 (12). The participants gave their written informed consent to voluntarily participate in this research, and their participation was based on their choice, free from the elements of assault, threat, injustice or manipulation ^18^; consent was not given to share their data ^19^.

### Recruitment

The reasons underlying the selection of the two public hospitals were firstly, that these hospitals provide HIV treatment and counselling to the patients and secondly, because the HIV patients from these hospitals are continuously engaging with the treatment. Moreover, these hospitals reported high numbers of HIV infected patients based on the yearly report of HIV statistics ^20^. In order to obtain information about the rich experiences of the participants, purposive sampling was used based on the HIV patients registered at the hospitals from February 2015 to February 2017. In March 2017, the medical staff at both selected hospitals helped by providing a list of names of PIWH. The selection of participants was based on inclusion and exclusion criteria. The inclusion criteria were being over 18 years old, having been infected with HIV for at least two years, and being literate. The exclusion criteria were being under 18 years old, having been infected with HIV for less than two years, and being illiterate.

Based on the inclusion and exclusion criteria, a total of 25 PIWH were identified as potential participants. An initial meeting was conducted with the potential participants to explain the purpose, ethical principles, and the duration of the study. The explanation of the research, which was conducted face-to-face and not part of the HIV treatment, was given by the researcher (TN) with the purpose of obtaining voluntary participation. The briefing session was conducted by the researcher and attended by the medical staff of each of the selected hospitals and the potential participants. Although the briefing session would disclose the HIV status of the participants to the researcher, who was not affiliated with the hospitals, the researcher was fully aware of the confidentiality required throughout this research ^21^.

### Participants

All 25 PIWH were willing to participate in this research. The first stage of the interview sessions was conducted with four voluntary PIWH who were selected based on their mode of HIV transmission; PWID, heterosexual, vertical transmission and MSM. The selection of participants based on mode of HIV transmission was helped by the medical staff in providing a list of names of PIWH. Throughout the study, a total of 12 PIWH participated in this research based on the data saturation ^22^, when no new categories or themes emerged from the interviews on the experiences of living with PIWH. The selection of 12 participants is consistent with grounded theory research, in which data gathering is carried out on datasets collected from 8–20 participants ^22, 23^.

### Procedure

This study used the grounded theory approach for data gathering. This approach identifies theories from data via logical assumption or inductive processes based on the observation and exploration of a phenomenon ^24^. It begins with the researcher identifying a research question that is broad, open ended, and action oriented ^22, 24^. In this study, it began with ‘How do PIWH experience life after being affected with HIV?’

Then, a group of people or settings that exemplify different facets of this question was chosen ^22^.

Semi-structured interviews, non-participant observations, and diary writing were involved in this study, with the main data provided by the semi-structured interviews. Semi-structured interviews involved one-to-one interaction between the researcher (TN) and the participants based on proposed protocols. Each of the interview sessions was conducted face-to-face between the participants and the researcher; a female and registered counsellor. The relationship between the researcher and the research participants started at the initial meeting, which was prior to the interview session. The participants were aware of the purpose of the research and the goals of the interview, which was to gather experiences as PIWH going through a counselling session. The researcher engaged in the interviews with the participants with prior knowledge of HIV and an interest in understanding the experiences of PIWH going about their lives. All the interview sessions were conducted in Bahasa Malaysia, which is the spoken language of the participants ^22^. All the interviews were audio recorded for the purpose of data gathering. Each of the interviews was conducted within one and half hours, and each of the participants was allowed time to respond to the open-ended questions to ensure they were covered in adequate depth ^21^. It was expected that the interviews would capture the key issues described by the research participants. Gathering this type of information requires flexibility and the use of semi-structured interviews is beneficial as it allows the participants to share their experiences using their own words and ways ^25^. The use of open-ended questions with the grounded theory approach as a guideline for the interviews was done to avoid assumptions ^18, 21, 23^.

Each of the participants had the chance to decide the date and time for the interview, which was decided according to their choice and was also free from the element of threat ^19^. All the interviews were conducted at the HIV clinic in the selected hospitals. Each participant participated in three interviews. The details of the interview questions for each of the three interviews are provided in Table 1. The interview questions were prepared prior to the actual data gathering, through preliminary interviews conducted with non-research participants before the actual research.

**Table 1.** The interview questions used in three rounds of interviews.

Interview number	Questions
First interview	1. How would you describe your background? 2. How would you describe your current situation? 3. What kind of feelings have you experienced since you became aware that you are HIV positive? 4. Tell me about your life after you discovered you are HIV positive. 5. How do you define yourself after you discovered you are HIV positive?
Second Interview	1. What changes have you faced since you discovered you are HIV positive? 2. Tell me about your experiences in facing the changes after you discovered you are HIV positive. 3. What are your feelings about facing the changes? 4. What can you tell me about the challenges you have faced since you discovered you are HIV positive? 5. What are your feelings about facing the challenges after you discovered you are HIV positive? 6. How do you see yourself in facing the challenges?
Third Interview	1. Tell me about your expectations about your life after being affected with HIV. 2. What are your ideas about your life since you discovered you are HIV positive? 3. What activities do you do since you discovered you are HIV positive? 4. What are your interests in life? 5. What are your feelings after having been through the treatment and counselling session? 6. What are your strengths?

This study also collected data by the observational method to describe the setting, activities, and people who participated in the study ^26^. Non-participant observations of the counselling session attended by the participants were performed in this study, each with a duration of one and half hours, and with each participant being observed at three different times after the interview sessions. The researcher (TN) visited the HIV clinic in the selected hospitals and conducted the observations without being involved in the counselling sessions between the medical staff and the participants ^21^.

Diary entries by the participants were also required as part of the data in this study as they represent a record of thoughts, feelings, opinions, or actions. The participants had the opportunity to share their experiences related to their lives as HIV infected individuals, actions, and personal information about themselves through the diaries ^27^. The diary entries allowed the researcher to compare the information shared by the participants during the interview and self-reported experiences written in the diary ^19^. Three diary entries were required for each participant during the study period. The participants were required to record their experiences in this study by completing pre-prepared statements ( Table 2), which were open-ended as this gives freedom to the participants to select their own words and style of writing ^27^. These diaries were completed by the participants at home.

**Table 2.** Pre-prepared statements used for diary entries.

No	Items
1	After engaging in the counselling session, my feelings are …..
2	During the counselling session, I am doing …..
3	My feelings during the activities ……
4	What I learn from this counselling session …..
5	My view on counselling services ……

### Data analysis

The study was conducted in Bahasa Malaysia, and the researcher (TN) transcribed all the interviews for the process of data analysis into English. The grounded theory approach was used in analysing the data, allowing categories, themes, and patterns to emerge ^23^. The stages of coding and the identification of the emergent themes was through the use of NVivo 8. The first stage of data analysis started immediately after the first data gathering process, with four initial interviews of PIWH from the different mode of HIV transmission. The initial analysis began with the first stage of data gathering, and analysis followed each interview, observation and diary writing thereafter. Then, through a process of constant comparison, the three main sources of data – interviews, non-participant observation and diary writing – were compared and contrasted with each other. Discussions among all the researchers at all stages of data analysis were conducted. Triangulation of the three main data and discussions among the researchers was to ensure the trustworthiness and credibility of the data analysis. The variety aspects of participants’ experiences with a different mode of HIV transmission increased the trustworthiness and the credibility of the findings that allowed for alternative interpretations ^26, 28^. Throughout all the stages of data collection and analysis, the researcher kept memos and drew diagrams. Details of the themes emerged the substantive theory that explained the experiences of PIWH can be seen in Table 3.

**Table 3.** Substantive theory of relationship and career challenges faced by PIWH.

Experiences of PIWH	**Main themes**	**Sub-themes**
	Career challenges	Difficult to get a job
		Career development is disrupted
	Relationship with others	Feeling uncomfortable
	Relationship with family	Rejection
		Labelling

## Results

A total of 12 participants contributed to the study, of which seven (58.3%) were male and five (41.7%) were female. Of these, three (25%) were PWID who contracted HIV via intravenous drug use, two (16.7%) contracted HIV via vertical transmission, four (33.3%) were heterosexual and contracted HIV via sexual transmission, and three (25%) were MSM who contracted HIV via sexual transmission. Overall, two (17%), three (25%) and seven (58%) of the participants were in the age groups <30 years old, 30–40 years old, and >40 years old, respectively. Regarding occupation, seven (58.3%) were employed, three (25%) were unemployed, and two (16.7%) were businessman. The summary of the background of the PIWH in the study is summarised in Table 4.

**Table 4.** Socio-demographic characteristics of participants.

Patient characteristics	Number	Percentage (%)
**Age (years)**		
<30	2	16.7
30–40	3	25
>40	7	58.3
**Gender**		
Male	7	58.3
Female	5	41.7
**Education**		
Secondary school	4	33.3
Certificate	4	33.3
Degree/Masters	4	33.3
**Occupation**		
Employed	3	25
Unemployed	7	58.3
Businessman	2	16.7
**Income (MYR)**		
<2000	5	41.7
2000–4000	4	33.3
>4000	3	25
**Mode of HIV transmission**		
PWID	3	25
Vertical	2	16.7
Sexual (heterosexual)	4	33.3
Sexual (MSM)	3	25
**Duration of HIV treatment (years)**		
<5	4	33.3
5–15	5	41.7
>15	3	25

MYR, Malaysian Ringgit; HIV, human immunodeficiency virus; PWID, people who inject drugs.

### Challenges of PIWH

The three main themes developed from the experiences of PIWH which related to, relationship with others, relationship with family and career challenges. Difficulties in coping with life and facing struggles to survive are among the challenges that cause negative feelings in affected people ^29, 30^. The PIWH in this study shared their experiences of facing life challenges in relation to their career as well as their relationship with family and others. The participants indicated that they had experienced career challenges in that it was difficult to get a job or advance their career. Besides that, the participants also shared the experience of having problems in their relationships with family and others. These challenges were found to be very much related to the mode of HIV transmission (intravenous drug use in PWID, vertical transmission, sexual transmission in MSM or heterosexual sexual transmission). The experiences shared by the research participants are outlined below.

### Career challenges

Career challenges underpinned the experiences among those infected with HIV via intravenous drug use, vertical transmission and heterosexual sexual transmission. Those infected via vertical transmission indicated that they experienced some difficulties in getting a job, while those infected via intravenous drug use and heterosexual sexual transmission experienced disruption to their career development.

***Difficult to get a job.*** One of the research participants infected via vertical transmission indicated that they faced difficulties in getting a job. According to Case 4 (aged 21, female), as HIV positive, her time was spent on regular treatment appointments, which made it impossible to think about getting a job. The participant was also tied by the way of life as an HIV positive individual:

*“After I finished my study, I started to plan for my future. I had a dream about a good job like others. But it remained a dream because I know that getting a job is not easy for HIV affected. I need to go to the hospital and have treatment regularly. Also, I have to be more careful about certain things like having healthy food and I need to stay alert to the changes happening in my body.”*

Another participant, Case 5 (aged 19, female), who was infected via vertical transmission also said that getting a job is impossible for PIWH. She had experienced being rejected as soon as the employer knew her status:

*“I am totally sure that getting a job is impossible for HIV affected. Before this, I had experience in applying for a job. I told the employer about my background and after he knew it, he said that I am not qualified to have the job.”*

The participants indicated that as HIV positive, they have no chance of getting a job because of their status.

***Career development disrupted.*** Being HIV positive can also disrupt career development, as experienced by those infected via intravenous drug use and heterosexual sexual transmission. According to Case 3 (aged 40, female), after being diagnosed with HIV, the participant started to lose motivation when working and missed opportunities to develop her career because of difficulties after being infected with HIV.

*“In working, I started to feel meaningless. I am good as a worker but after being diagnosed with HIV I cannot focus properly on my work. I need to go to the hospital to get treatment, and I think I spend more time in hospital than the workplace. Frequently, I was not feeling well because of HIV. I also started to miss the opportunities to get a bonus from the company.”*

Another participant who was infected via intravenous drug use, Case 2 (aged 51, male), mentioned in his diary that HIV had raised some barriers in his working life. The barriers were related to expanding his business and making more profit.

*“It is really difficult to live with HIV. It gives me more stress, especially in working. After the diagnosis, I started to have some struggles in expanding my work. I did not always feel well, and am totally dependent on medicines because of my CD4 getting lower. Now, I am unable to work overtime and cannot focus on my work.”*

In addition, during the non-participant observation, Case 2 verbally indicated to the medical staff that he was worried about his business situation because of the amount of time he spent in the hospital for treatment.

Another participant who was infected via heterosexual sexual transmission, Case 7 (aged 42, female), said that as HIV affected, she started to have some problems in her working life. The participant said that her work was disrupted when she had some emotional struggles in facing her HIV infection. The difficulties in handling these emotions affected her career development.

*“Only God knows how I felt during the HIV diagnosis. I was totally out of myself and I planned to quit my job. At the same time, I was offered a salary increment. I just did not know what was the best thing to do. I was at a loss.”*

Another participant who was infected via heterosexual sexual transmission, Case 6 (aged 48, female), also stated in her diary that HIV infection made her life worse as her business was almost ruined due to her situation. The participant needed to focus on her business, while, at the same time, she was struggling to adhere to the treatment regularly and sometimes she got depressed about continuing the treatment.

*“Every day I kept thinking about my business. This business should be continued even if I am not feeling well. I had to be strong to make my business run smoothly. Sometimes I was really afraid that my condition would not allow me to focus on it.”*

The participants indicated that HIV infection is a barrier to them developing their career. This was related to challenges in dealing with HIV infection as the participants had to deal with regular treatment and the daily activities as HIV infected.

### Relationship with others

The participants also reported that HIV affected their relationship with others. This was experienced by those infected via heterosexual sexual transmission and MSM who were infected via sexual transmission. The participants started to feel uncomfortable about being with others and chose to isolate themselves from others.

***Feeling uncomfortable.*** The participants infected via heterosexual sexual transmission indicated that they felt uncomfortable being with others as they wanted to ensure that their status would not be disclosed. Case 7 shared the uncomfortable feeling of being with others and stated that the feeling was related to the changes in her physical appearance after being infected with HIV.

*“When I knew the result of my HIV status, I noticed that I started to feel awkward being with people, especially my friends. I did not want them to know my HIV status. I changed my routine and I just spent more time with my children at home. Besides that, I felt uncomfortable about the physical changes as I became thinner and my skin looked so dull.”*

In addition, in her diary, Case 7 mentioned that she was not really comfortable about attending the treatment sessions in hospital if there were other patients in the same session.

*“During the treatment, I was not comfortable if I had to be with a lot of patients in the same session. I was worried that someone would notice me attending HIV treatment in the hospital.”*

Another participant who was infected via sexual transmission, Case 6, also shared the feeling of being uncomfortable since being affected with HIV. It changed her a lot, especially her physical appearance, and she worried about facing people when her physical appearance was different.

*“With HIV, I looked so dull. I was so particular in my appearance as a businesswoman. I have to face and meet people in my career. The treatment affected me a lot. I was so not comfortable with the medicine I was taking. The medicine had some effect, especially in terms of my physical appearance.”*

The participants indicated that the feeling of being uncomfortable affected their life regarding their relationships with others. In addition, the feeling was also related to the changes in their physical appearance as the participants faced people in their daily life.

***Denial.*** The participants also reported being in denial about being HIV positive. This was experienced by those infected via heterosexual sexual transmission and MSM who were infected via sexual transmission. Case 10 (aged 36, male) said that it was difficult to accept being infected with HIV. This was because the participant realized that his wife could not accept the situation.

*“When I received the HIV result, the first thing that came into my mind was my wife. I couldn’t imagine how she would react towards this matter. I just could not accept this.”*

Another MSM participant, Case 11 (aged 44, male), also indicated about being in denial. The participant said that it was very hard to accept the reality of being affected with HIV.

*“It was so unbelievable. I was a very particular person in any matter related to myself, especially my health. I felt like it was not me. I tried to think positive, but I knew this was the reality. It was so hard for me.”*

In addition, Case 10 mentioned in his diary that he would never accept that he was HIV positive.

*“Even for thousand years upward, I would never accept HIV is inside my body.”*

Another MSM participant, Case 12 (aged 45, male), also said that it was very hard to accept the reality of being infected with HIV.

“ *I prayed that it was not me. I hoped the result was not mine. I repeated the test about three times just to make sure that the result was true. It hurts me.”*

The participants indicated experiencing a feeling of being uncomfortable with others and denial in terms of accepting the fact that they are HIV positive. These experiences also contributed to the struggles faced in their daily life.

### Relationship with family

The participants also indicated having challenges in their relationships with their families. They had experienced rejection and labelling from the family members.

***Rejection.*** Being HIV affected, Case 10 said that he could never face his family since he experienced rejection from his mother and siblings.

*“I never informed my family about my HIV status. One day, my sister came into my room and she found the documents of treatment on my bed. Then, the news was spread. The situation was so tense. Then I knew that no one in my family would accept this.”*

Case 10 also mentioned in his diary that he would never put on his family the burden of living with a HIV affected person.

*“I can’t even manage my feelings when I see my mother crying. All I can say is that this burden (HIV affected) should never be laid on them (family).”*

***Labelling.*** Participants infected via heterosexual transmission and MSM who were infected by sexual transmission reported that they were also labelled by their family members as HIV positive. Case 7 stated that she had experienced being labelled by her siblings.

*“I have experienced this, my brother came to me and said it to my face that I have HIV. So, he said that the family need to be more careful in sharing the things in the house with me.”*

In addition, Case 11 also said that his family labelled him as being disgusting, dirty, and irritating in the eyes of others, including his siblings.

*“When they asked about the plate I had used, or when they asked which plate I had used, it clearly showed that they were not really happy about it.”*

In summary, their experiences in facing life challenges caused changes in the reality of life for PIWH. Being HIV positive, they need some space or area to be accepted and heard. Their sharing of these experiences showed that these challenges were experienced when they were engaging with others or being in public.

## Discussion

This study provides knowledge of the challenges experienced by PIWH. These challenges have been found to influence PIWH’s relationships with family and others and affect their careers. HIV could have a severe impact on the career opportunities ^31^ and the stability of relationships ^32^. This can cause social isolation and conflictual social interactions that may increase stress, resulting in poorer overall social functioning ^33^. In this study, it was found that the challenges experienced by PIWH were different based on the mode of HIV transmission among PWID who were infected via intravenous drug use, those infected via vertical transmission, those infected via heterosexual sexual transmission, and those infected by homosexual sexual transmission. Although the experiences of PIWH may be similar in certain ways, the way they see the experiences could be different based on their background as HIV infected individuals.

The participants reported that career development was among the challenges faced by them. This was experienced by PWID and those infected via vertical transmission and heterosexual sexual transmission. They struggled to get a job and found it was very difficult to continue their career once they started to notice changes after being affected with HIV. The reasons for these experiences were related to them having to concentrate on regular treatment, needing an adjustment in terms of arranging the schedule for treatments and struggling to concentrate on their work. These findings are related to the view ^34^ that PWID infected with HIV are concerned about being different due to the need to take medication throughout the day and the decision of whether or not to confide in their supervisors and co-workers about the reason why they are frequently taking this medication. Besides that, these feelings may lead PIWH to decrease their achievements, classify their lives as not important, and resist recognizing their long-term goals ^35^. For example, Case 2 and Case 3 reported that it was very difficult to perform well in their career after changes to their routine since being HIV positive, as they needed to focus on the treatment provided. Therefore, the findings of this study can be related to Adler’s concept of social interest, as the problems of the individual are related to the feeling of being unaccepted in their social community ^12^. Hence, the participants need to have some support system that can help them to adapt to a new routine of life after being infected with HIV.

This study also indicated that those who were infected via heterosexual sexual transmission, and MSM who were infected by sexual transmission struggled to maintain their relationships with others. For example, Case 7 was not comfortable being with others. The participant became worried about not being accepted if their HIV status was disclosed to other people. This can be seen from the Adlerian perspective as human behaviour is determined by the capacity to interpret the events according to the social interest concept ^12^. In addition, Adler stated that people express social interest through shared activity, cooperation, participation in the common good, and mutual respect. With regard to Case 7, which explained about the uncomfortable feeling when being with others was based on the thinking that no one would accept the reality of her HIV status. Besides that, the participants also reported denial of the situation. For example, Case 9 stated that it was very hard to accept the fact that they were infected with HIV. According to the participant, this feeling was related to feelings of worry about his spouse if his HIV status was known. This is because the disease not only affects the patient, but also their family members, especially their spouse ^10^. In other words, HIV status creates challenges not only with others but also a personal dilemma among those who are HIV affected.

The participants who had been infected via heterosexual sexual transmission and MSM who were infected by sexual transmission also reported challenges in facing their family. Under the category of relationships with family, the most important issues raised by participants were rejection and labelling from family members. In relation to this, appropriate support from families enables HIV infected MSM to have appropriate responses to the stress caused by illness, and, therefore, they would have fewer mental health issues ^10^. For example, Case 8 stated that he chose not to see his family when he noticed their rejection and showed that he was stressed from the experience of rejection from the family. This situation can be seen through the Adlerian perspective; an individual seeks for supports in relationship with the family and society to secure their need for security, acceptance, and worthiness ^36^. Moreover, Case 7 also experienced being labelled by family members, which contributed to their feelings of isolation. In relation to this, when families and society treat patients negatively, so that they are isolated and excluded when found to be HIV positive, the disease will spread in a clandestine mode of transmission and a major part of the infected population will remain hidden ^37– 40^. There is a limitation to the study. Trust relationship need to be developed prior the data gathering process among the participants before engaging with interviews. The findings cannot be generalized to broader populations of PIWH with the same degree of certainty. This is because the findings of this study were not tested to determine whether they were statistically important or due to chance.

## Conclusions

The experiences of PIWH indicated that their career challenges and uncomfortable relationships with other people, lead to physical and mental health issues, such as physical pain and emotional struggles. In the study, the participants of HIV infected via sexual transmission were found to have more struggles in their lives compared to other participants from another mode of HIV transmission. Their experiences of these challenges can affect their daily lives as human beings. Even though the rates of HIV infection have already declined, the findings showed that PIWH still needed help to handle the challenges of living with HIV. Therefore, knowledge and awareness about HIV prevention among the community, non-governmental organisations and HIV treatment providers should be highlighted by the policymakers. Policies concerning the prevention of HIV need to be improved and can be emphasized in collaboration with government agencies to ensure the success of these prevention programmes.

These findings also have important implications for counselling services for HIV/AIDS prevention in Malaysia as well as in similar settings in other low- and middle-income countries. There is a need for further similar research to be conducted, particularly counselling services for HIV infected people.

## Data availability

### Underlying data

The underlying data for this study cannot be openly shared since the consent to participate obtained from the HIV patients explicitly stated that their data would remain confidential and only be reported in an aggregated manner. Anyone wishing to access the underlying data should first contact the corresponding author ( ruhani@umt.edu.my), who will facilitate contact with the ethical review board that approved the study. Access will be granted upon approval from the ethical review board.
