# Supplementary material for: Relationship and career challenges faced by people infected with HIV in Malaysia
Source: F1000Res. 2020 Oct 23;8:1994. Originally published 2019 Nov 26. [Version 3] doi: 10.12688/f1000research.21079.3 (PMC7649721; doi:10.12688/f1000research.21079.3)
Supplement: Peer review history

# Peer review: Reviewer response for version 3

**DOI:** 10.5256/f1000research.30348.r73618

**Published:** 2020-11-06

**Authors:** Kristina Lindvall

**Affiliations:** 1 Department of Epidemiology and Global Health, Umeå University, Umeå, Sweden

Thank you to the Editor and the Authors for the additional opportunity to read the article. It is very interesting. I have no additional comments on the revised article. The revisions made by the authors are sufficient.

Is the work clearly and accurately presented and does it cite the current literature?

Yes

If applicable, is the statistical analysis and its interpretation appropriate?

Not applicable

Are all the source data underlying the results available to ensure full reproducibility?

Partly

Is the study design appropriate and is the work technically sound?

Yes

Are the conclusions drawn adequately supported by the results?

Yes

Are sufficient details of methods and analysis provided to allow replication by others?

Partly

Reviewer Expertise:

Public Health, Nutrition, Qualitative methodology

I confirm that I have read this submission and believe that I have an appropriate level of expertise to confirm that it is of an acceptable scientific standard.

# Peer review: Reviewer response for version 3

**DOI:** 10.5256/f1000research.30348.r73617

**Published:** 2020-10-26

**Authors:** Syed Mohamed Aljunid

**Affiliations:** 1 Department of Health Policy and Management, Faculty of Public Health, Kuwait University, Kuwait City, Kuwait; 2 International Centre for Casemix and Clinical Coding, Universiti Kebangsaan Malaysia Medical Centre, Kuala Lumpur, Malaysia

The authors have addressed all the comments and questions asked in this paper. I am satisfied with their responses and the paper can be accepted for publication.

Is the work clearly and accurately presented and does it cite the current literature?

Yes

If applicable, is the statistical analysis and its interpretation appropriate?

Not applicable

Are all the source data underlying the results available to ensure full reproducibility?

Partly

Is the study design appropriate and is the work technically sound?

Yes

Are the conclusions drawn adequately supported by the results?

Yes

Are sufficient details of methods and analysis provided to allow replication by others?

Partly

Reviewer Expertise:

NA

I confirm that I have read this submission and believe that I have an appropriate level of expertise to confirm that it is of an acceptable scientific standard.

# Response

**Published:** 2020-10-26

**Authors:** Tuan Norbalkish Tuan Abdullah

**Affiliations:** University Malaysia Terengganu, Malaysia

Thank you for your positive feedback. We are grateful for your time and appreciate your comments on this manuscript.

# Peer review: Reviewer response for version 2

**DOI:** 10.5256/f1000research.29300.r70974

**Published:** 2020-10-06

**Authors:** Syed Mohamed Aljunid

**Affiliations:** 1 Department of Health Policy and Management, Faculty of Public Health, Kuwait University, Kuwait City, Kuwait; 2 International Centre for Casemix and Clinical Coding, Universiti Kebangsaan Malaysia Medical Centre, Kuala Lumpur, Malaysia

I found that the authors have not addressed any of my comments adequately. Please ask the authors to provide 'point-by-point" response to my comments.

Is the work clearly and accurately presented and does it cite the current literature?

Yes

If applicable, is the statistical analysis and its interpretation appropriate?

Not applicable

Are all the source data underlying the results available to ensure full reproducibility?

Partly

Is the study design appropriate and is the work technically sound?

Yes

Are the conclusions drawn adequately supported by the results?

Yes

Are sufficient details of methods and analysis provided to allow replication by others?

Partly

Reviewer Expertise:

NA

I confirm that I have read this submission and believe that I have an appropriate level of expertise to confirm that it is of an acceptable scientific standard, however I have significant reservations, as outlined above.

# Response

**Published:** 2020-10-07

**Authors:** Tuan Norbalkish Tuan Abdullah

**Affiliations:** University Malaysia Terengganu, Malaysia

We would like to thank the reviewer for pointing out insightful comments and we are also apologize for the inconvenience.

 1) Methods: On page 4, right column para1, the authors stated that they used the open-ended questionnaire with grounded theories to avoid assumptions. Please elaborate on the kind of assumptions that they want to avoid in this study

 Answer: This study conducted open-ended semi-structured interviews, which provides the participants free elements in giving their answers.

 2) Methods: On page 4, right column para 2: it was mentioned that the date and time of interview were selected by respondents to avoid "threat".  Please elaborate what kind of threat are they concerned about since the interviews were carried out in the clinics.

 Answer: Here, the participants had their autonomy which they were free from any threat elements like being forced to be involved in the study.

 3) Methods: Regarding Diaries entries, what are the authors expectation on the respondents entrance of the diaries? What kind of guidances were given to the respondents on what to record in the diary.

 Answer: Table 2: Pre-prepared statements used for diary entries.

 4) Explain how items in Table 2 on page 5 were used in this study to guide the respondents?

 Answer: Table 2: Pre-prepared statements used for diary entries: By completing this pre-prepared statements (stated in the document)

 5) Results: What do you mean by vertical transmission here? Are you sure this information is accurate? Who gave this information?  Was it from the respondents or the doctors who treat the patients? How do you verify this information

 Answer: The term of vertical is verified by the medical staff who were involved in the selection of participants. ( Participants).

 6) Discussion: Page 9, first line on right column: Clarify what is the meaning of "feeling felt"?

 Answer: Agreed and revised.

 7) Discussion: Page 9, right column, 2nd paragraph: Please explain in more detail how Adlerian perspective is relevant to the findings of this research.

 Answer: Agreed and revised.

 8) Conclusion: Please revise the first sentence of the Conclusion. It is not clear what the authors trying to say here.

 Answer: Agreed and revised.

# Peer review: Reviewer response for version 2

**DOI:** 10.5256/f1000research.29300.r70973

**Published:** 2020-09-14

**Authors:** Kristina Lindvall

**Affiliations:** 1 Department of Epidemiology and Global Health, Umeå University, Umeå, Sweden

I would like to thank the Editor and the authors for the opportunity to read the revised version of the manuscript. The authors have taken careful consideration of my comments. I still have a few more comments remaining since the first version and a few that are added based on the revision. I have referred to the section and heading below in relation to each comment.

**1. Methods: Under the heading ”participants”**

 Suggest reformulating the new sentence;

 “Throughout the study, a total of 12 PIWH participated in this research based on the data saturation ^22^ , when there were no more new ideas existed from the experiences which involved different types of HIV transmission as PWID, heterosexual, MSM or vertical transmission”.

 to either:

 Throughout the study, a total of 12 PIWH participated in this research based on the data saturation ^22^ , when no new ideas emerged from the interviews on the experiences of living with PIWH.

 Or:

 Throughout the study, a total of 12 PIWH participated in this research based on the data saturation ^22^ , when no new categories or themes emerged from the analysis of the interviews on experiences of living with PIWH.

**2. **D**ata analysis (trustworthiness).** I still wonder if the authors used any of the other criteria than credibility (for example transferability, dependability or confirmability) to ensure trustworthiness? Below is a doi and a link to an article by Graneheim and Lundman describing some of these criteria (also referring to GT criteria). Link: https://www.sciencedirect.com/science/article/pii/S0260691703001515?via%3Dihub ^1^

 Doi: doi: 10.1016/j.nedt.2003.10.001.

**3. **F**igure 1.** This figure has been changed since the revision. I still think it would be helpful and important with a description in the figure text on for example what the arrows and the boxes represent and what level of analysis it is we see in the boxes (i.e is it for example categories, themes, and/or codes)

**4.** **Results.** Heading “Challenges with PIWH”. The authors describe two main themes in the summary of the results, i.e. “Career challenges” and “Relationship”. But in the text I believe I see three themes (if following the headings). I.e Career challenges, Relationship with others and Relationship with family. Would it be possible to clarify this under the heading "Challenges with PIWH".

**5. Conclusion.** I would suggest the authors to consider moving the following sentences in the conclusion to last in the discussion. "There is a limitation to the study. Trust relationships need to be developed prior to the data gathering process among the participants before engaging with interviews. The findings cannot be generalized to broader populations of PIWH with the same degree of certainty. This is because the findings of this study were not tested to determine whether they were statistically important or due to chance"

Is the work clearly and accurately presented and does it cite the current literature?

Yes

If applicable, is the statistical analysis and its interpretation appropriate?

Not applicable

Are all the source data underlying the results available to ensure full reproducibility?

Partly

Is the study design appropriate and is the work technically sound?

Yes

Are the conclusions drawn adequately supported by the results?

Yes

Are sufficient details of methods and analysis provided to allow replication by others?

Partly

Reviewer Expertise:

Public Health, Nutrition, Qualitative methodology

I confirm that I have read this submission and believe that I have an appropriate level of expertise to confirm that it is of an acceptable scientific standard, however I have significant reservations, as outlined above.

## References

[1] : Qualitative content analysis in nursing research: concepts, procedures and measures to achieve trustworthiness. *Nurse Educ Today*.2004;24(2) : 10.1016/j.nedt.2003.10.001 105-12 10.1016/j.nedt.2003.10.001 14769454

# Response

**Published:** 2020-10-20

**Authors:** Tuan Norbalkish Tuan Abdullah

**Affiliations:** University Malaysia Terengganu, Malaysia

We would like to thank you for the constructive comments in this review. 

1. Methods

Answer: Agreed and revised.

2. Data Analysis

Answer: Agreed and revised

3. Figure

Answer: Agreed and revised

4. Result

Answer: Agreed and revised

5. Conclusion

Answer: Agreed and revised

# Peer review: Reviewer response for version 1

**DOI:** 10.5256/f1000research.23198.r68394

**Published:** 2020-08-17

**Authors:** Kristina Lindvall

**Affiliations:** 1 Department of Epidemiology and Global Health, Umeå University, Umeå, Sweden

I want to thank the authors and the Editor of F1000 Research for the opportunity to review this interesting manuscript.

 Below are my comments alternatively suggestions for revision divided into the sections in which they appear.

**Abstract:**

1. The background of the abstract does not include any motivation for the need of this study. Instead, it describes the setting and the aim of the study. If the word limit allows I would suggest adding one sentence motivating the need of the study. If there is a need to save words I would suggest deleting the first sentence in the abstract alternatively some of the details in the methods. For example, to end the sentence starting with “The interviews and diaries were transcribed… after the word “approach”.

**Introduction (Aim):**

1. I would suggest using the same aim in the abstract as in the main manuscript. Now they are slightly different (for example in the abstract the authors mention “coping”). I would also suggest either using the word “aim” or “purpose” in both the abstract and the main text for consistency. 

**Methods:**

1. First paragraph: Would it be possible to add where in the country the two hospitals are situated, alternatively how large areas of the country that these hospitals cover?
2. Paragraph labelled “Participants”: The authors mention that the final number of participants was decided based on saturation. Would it be possible to add some more information on how you determined that you had reached saturation (either in this paragraph or in “data analysis”)?
3. “Procedure”, fifth paragraph: The authors describe, “This study also collected data by the observational method to describe the setting, activities, and people who participated in the study”. Where can the reader see the results of these observations?
4. Data analysis: The authors describe, “The first stage of data analysis started immediately after the first data gathering process”. Which “firs data gathering process” is referred to here? For example, does it mean that data analysis started after the first one or two interviews, after all interviews had been conducted or something different?
5. Data analysis: Did the authors use initial coding and focused coding during the analytical process? If yes, I suggest adding this to the “data analysis” paragraph. I would also suggest using the same terminology in the methods and in the results. I.e if categories and themes and/or sub-categories are mentioned in the results then I believe it would be helpful if these are also referred to in the analytical process described in the paragraph “data analysis”.
6. Data analysis: Did the authors start to analyze one source of material (for example the interviews) and then continued with the next or were all three sources (interviews, observations, diary entries) analyzed simultaneously.
7. Data analysis (Trustworthiness): Were there any other criteria used to ensure trustworthiness than credibility (for example, transferability, dependability confirmability)? If, yes. It would be helpful if the authors could add some more information in relation to this.
8. Table 1: One alternative table heading could be “Interview questions used in three rounds of interviews”.
9. Table 2: One alternative table heading could be “Pre-prepared statements used for diary entries”.
10. Figure 1. Could the authors please add some more detail to the figure text? For example, where can the reader see the data collection in the figure? What do the arrows, lines and squares in the figures represent? If some squares represent participants and others, for example represent the results. Could these differences be indicated by squares being different in the frame, shape or shading? 

**Results:**

1. First paragraph: I wonder if it perhaps is enough with one decimal for the percentages presented?
2. First paragraph: If possible, it would be interesting to also the age range of participants.
3. Did the authors collect any information on if the participants lived in a more urban or rural setting alternatively in a smaller or larger city? If this is available, perhaps this could be added to table 3.
4. Just after the first paragraph of the results and before the paragraph with the heading “Challenges of PIWH” it would be helpful if the authors could add an introduction of the results. I.e how many themes or categories that emerged and how many sub-categories (depending on how the authors prefer to label these) that emerged and which these were. In this way, the reader is also helped to see what the headings and the sub-headings of the results represent.

**Discussion:**

1. First paragraph: Here the authors state that “In this study, it was found that the challenges experienced by PIWH *were different based on the mode of HIV transmission among PWID* who were infected via intravenous drug use, those infected via vertical transmission, those infected via heterosexual sexual transmission, and those infected by homosexual transmission”. Here it would be helpful if the authors could add one or two sentences summarizing these differences.
2. Second paragraph. Do the authors see any limitations with the current study being based on a *qualitative* study design? To me, the limitation currently raised more to the study not having a quantitative study design.

**Conclusions:**

1. It would be helpful if the authors could highlight what they find as their main findings in the first two-three sentences of the conclusion.

Is the work clearly and accurately presented and does it cite the current literature?

Yes

If applicable, is the statistical analysis and its interpretation appropriate?

Not applicable

Are all the source data underlying the results available to ensure full reproducibility?

Partly

Is the study design appropriate and is the work technically sound?

Yes

Are the conclusions drawn adequately supported by the results?

Yes

Are sufficient details of methods and analysis provided to allow replication by others?

Partly

Reviewer Expertise:

Public Health, Nutrition, Qualitative methodology

I confirm that I have read this submission and believe that I have an appropriate level of expertise to confirm that it is of an acceptable scientific standard, however I have significant reservations, as outlined above.

# Response

**Published:** 2020-09-03

**Authors:** Tuan Norbalkish Tuan Abdullah

**Affiliations:** University Malaysia Terengganu, Malaysia

Abstract: The background of the abstract does not include any motivation for the need of this study. Instead, it describes the setting and the aim of the study. If the word limit allows I would suggest adding one sentence motivating the need of the study. If there is a need to save words I would suggest deleting the first sentence in the abstract alternatively some of the details in the methods. For example, to end the sentence starting with “The interviews and diaries were transcribed… after the word “approach”.

Answer: Agreed with your comments and already revised

Introduction:     I would suggest using the same aim in the abstract as in the main manuscript. Now they are slightly different (for example in the abstract the authors mention “coping”). I would also suggest either using the word “aim” or “purpose” in both the abstract and the main text for consistency

Answer: Thank you for suggestions

Methods: 1.       First paragraph: Would it be possible to add where in the country the two hospitals are situated, alternatively how large areas of the country that these hospitals cover?

Answer: This is related to the ethical issue, which in Malaysia, there are only general hospitals from each state that manage HIV cases.

2.            Paragraph labelled “Participants”: The authors mention that the final number of participants was decided based on saturation. Would it be possible to add some more information on how you determined that you had reached saturation (either in this paragraph or in “data analysis”)?

Answer: Agreed with your comments and already revised

3.            “Procedure”, fifth paragraph: The authors describe, “This study also collected data by the observational method to describe the setting, activities, and people who participated in the study”. Where can the reader see the results of these observations?

Answer: Agreed with your comments and already revised

4.            Data analysis: The authors describe, “The first stage of data analysis started immediately after the first data gathering process”. Which “firs data gathering process” is referred to here? For example, does it mean that data analysis started after the first one or two interviews, after all interviews had been conducted or something different?

 Answer: Agreed with your comments and already revised

5.            Data analysis: Did the authors use initial coding and focused coding during the analytical process? If yes, I suggest adding this to the “data analysis” paragraph. I would also suggest using the same terminology in the methods and in the results. I.e if categories and themes and/or sub-categories are mentioned in the results then I believe it would be helpful if these are also referred to in the analytical process described in the paragraph “data analysis”.

Answer: Agreed with your comments and already revised

6.            Data analysis: Did the authors start to analyze one source of material (for example the interviews) and then continued with the next or were all three sources (interviews, observations, diary entries) analyzed simultaneously.

Answer: Agreed with your comments and already revised

7.            Data analysis (Trustworthiness): Were there any other criteria used to ensure trustworthiness than credibility (for example, transferability, dependability confirmability)? If, yes. It would be helpful if the authors could add some more information in relation to this.

 Answer: Triangulation

8.            Table 1: One alternative table heading could be “Interview questions used in three rounds of interviews”.

 Answer: Agreed with your comments and already revised

9.            Table 2: One alternative table heading could be “Pre-prepared statements used for diary entries”.

 Answer: Agreed with your comments and already revised

10.          Figure 1. Could the authors please add some more detail to the figure text? For example, where can the reader see the data collection in the figure? What do the arrows, lines and squares in the figures represent? If some squares represent participants and others, for example represent the results. Could these differences be indicated by squares being different in the frame, shape or shading?

Answer: Agreed with your comments and already revised

Results: 1.           First paragraph: I wonder if it perhaps is enough with one decimal for the percentages presented?

Answer: Agreed with your comments and already revised

2.            First paragraph: If possible, it would be interesting to also the age range of participants

Answer: It is already stated in the table the range of participants

3.            Did the authors collect any information on if the participants lived in a more urban or rural setting alternatively in a smaller or larger city? If this is available, perhaps this could be added to table 3.

 Answer:  The information of the participants was not involved/considered of urban and rural setting.

4.            Just after the first paragraph of the results and before the paragraph with the heading “Challenges of PIWH” it would be helpful if the authors could add an introduction of the results. I.e how many themes or categories that emerged and how many sub-categories (depending on how the authors prefer to label these) that emerged and which these were. In this way, the reader is also helped to see what the headings and the sub-headings of the results represent.

Answer: Agreed with your comments and already revised

Discussion: 1.     First paragraph: Here the authors state that “In this study, it was found that the challenges experienced by PIWH were different based on the mode of HIV transmission among PWID who were infected via intravenous drug use, those infected via vertical transmission, those infected via heterosexual sexual transmission, and those infected by homosexual transmission”. Here it would be helpful if the authors could add one or two sentences summarizing these differences.

Answer: Agreed with your comments and already revised 

2.            Second paragraph. Do the authors see any limitations with the current study being based on a qualitative study design? To me, the limitation currently raised more to the study not having a quantitative study design.

Answer: Agreed with your comments and already revised (Conclusion Para 2)

Conclusion: It would be helpful if the authors could highlight what they find as their main findings in the first two-three sentences of the conclusion.

Answer: Agreed with your comments and already revised

# Peer review: Reviewer response for version 1

**DOI:** 10.5256/f1000research.23198.r66794

**Published:** 2020-08-10

**Authors:** Syed Mohamed Aljunid

**Affiliations:** 1 Department of Health Policy and Management, Faculty of Public Health, Kuwait University, Kuwait City, Kuwait; 2 International Centre for Casemix and Clinical Coding, Universiti Kebangsaan Malaysia Medical Centre, Kuala Lumpur, Malaysia

This is an excellent paper reporting on the outcome of qualitative research carried out on HIV positive patients to understand the challenges they face in life. The authors have reported the details of their encounter with 12 respondents using a semi-structured questionnaire in a face-to-face interview, non-participant observations, and respondents' diaries. There are a number of issues as follows that the authors need to address in order to strengthen the paper for the final submission:

1. Methods: On page 4,  right column para1, the authors stated that they used the open-ended questionnaire with grounded theories to avoid assumptions. Please elaborate on the kind of assumptions that they want to avoid in this study
2. Methods: On page 4, right column para 2: it was mentioned that the date and time of interview were selected by respondents to avoid "threat".  Please elaborate what kind of threat are they concerned about since the interviews were carried out in the clinics.
3. Methods; Regarding Diaries entries, what are the authors expectation on the respondents entrance of the diaries? What kind of guidances were given to the respondents on what to record in the diary.
4. Methods: Explain how items in Table 2 on page 5 were used in this study to guide the respondents?
5. Results: What do you mean by vertical transmission here? Are you sure this information is accurate? Who gave this information?  Was it from the respondents or the doctors who treat the patients? How do you verify this information
6. Discussion: Page 9, first line on right column: Clarify what is the meaning of "feeling felt"?
7. Discussion: Page 9, right column, 2nd paragraph: Please explain in more detail how Adlerian perspective is relevant to the findings of this research.
8. Conclusion: Please revise the first sentence of the Conclusion. It is not clear what the authors trying to say here.

Is the work clearly and accurately presented and does it cite the current literature?

Yes

If applicable, is the statistical analysis and its interpretation appropriate?

Not applicable

Are all the source data underlying the results available to ensure full reproducibility?

Partly

Is the study design appropriate and is the work technically sound?

Yes

Are the conclusions drawn adequately supported by the results?

Yes

Are sufficient details of methods and analysis provided to allow replication by others?

Partly

Reviewer Expertise:

Public Health Medicine and Health Policy and Management

I confirm that I have read this submission and believe that I have an appropriate level of expertise to confirm that it is of an acceptable scientific standard, however I have significant reservations, as outlined above.

# Response

**Published:** 2020-09-03

**Authors:** Tuan Norbalkish Tuan Abdullah

**Affiliations:** University Malaysia Terengganu, Malaysia

Comments

Respond to the review

Methods: On page 4, right column para1, the authors stated that they used the open-ended questionnaire with grounded theories to avoid assumptions. Please elaborate on the kind of assumptions that they want to avoid in this study

This study conducted open-ended semi-structured interviews.

Methods: On page 4, right column para 2: it was mentioned that the date and time of interview were selected by respondents to avoid "threat".  Please elaborate what kind of threat are they concerned about since the interviews were carried out in the clinics.

Open-ended semi structured interviews

Methods: Regarding Diaries entries, what are the authors expectation on the respondents entrance of the diaries? What kind of guidances were given to the respondents on what to record in the diary.

see document

Explain how items in Table 2 on page 5 were used in this study to guide the respondents?

See document

Results: What do you mean by vertical transmission here? Are you sure this information is accurate? Who gave this information?  Was it from the respondents or the doctors who treat the patients? How do you verify this information

See document

Discussion: Page 9, first line on right column: Clarify what is the meaning of "feeling felt"?

See document

Discussion: Page 9, right column, 2nd paragraph: Please explain in more detail how Adlerian perspective is relevant to the findings of this research.

See document

Conclusion: Please revise the first sentence of the Conclusion. It is not clear what the authors trying to say here.

See document
